# Homozygosity for a Clinically Significant *GALC* Haplotype Associated with Late-Infantile Krabbe Disease Detected on Newborn Screening: Implications for Clinical Management and Genetic Counseling

**DOI:** 10.3390/ijns12030059

**Published:** 2026-07-29

**Authors:** Daniel R. Schecter, Colleen Donnelly, Amy White, Hillary Raynes, Gordon Heller, Deepa Rajan, Carlos A. Saavedra-Matiz, Joseph Orsini, Jaap-Jan Boelens, Dietrich Matern, Jaya Ganesh

**Affiliations:** 1 Department of Medical Genetics and Genomic Sciences, Icahn School of Medicine at Mount Sinai, New York, NY 10029, USA; daniel.schecter@mountsinai.org (D.R.S.);; 2 Center for AI in Children’s Health, Mindich Child Health and Development Institute, Icahn School of Medicine at Mount Sinai, New York, NY 10029, USA; 3 Division of Laboratory Genetics and Genomics, Department of Laboratory Medicine and Pathology, Mayo Clinic College of Medicine and Science, Rochester, MN 55905, USA; white.amy1@mayo.edu (A.W.);; 4 Department of Pediatrics, Icahn School of Medicine at Mount Sinai, New York, NY 10029, USA; 5 Department of Neurology, Icahn School of Medicine at Mount Sinai, New York, NY 10029, USA; 6 Department of Radiology, Icahn School of Medicine at Mount Sinai, New York, NY 10029, USA; 7 Department of Pediatrics, UPMC Children’s Hospital of Pittsburgh, University of Pittsburgh School of Medicine, Pittsburgh, PA 15213, USA; 8 Newborn Screening Program, Wadsworth Center, New York State Department of Health, Albany, NY 12203, USA; 9 Transplantation and Cellular Therapies, Memorial Sloan Kettering Cancer Center, New York, NY 10065, USA; 10 Department of Pediatrics, Weill Cornell Medical College, Cornell University, New York, NY 10065, USA

**Keywords:** Krabbe disease, GALC, galactosylceramidase, newborn screening, hematopoietic stem cell transplant

## Abstract

Krabbe disease is an autosomal recessive leukodystrophy caused by a deficiency of the lysosomal enzyme galactosylceramidase (GALC), responsible for the degradation of galactolipids, resulting in toxic psychosine accumulation and progressive demyelination of the central and peripheral nervous systems. Newborn screening (NBS) has increased recognition of later-onset Krabbe disease, although interpretation of complex *GALC* genotypes remains challenging, particularly in the presence of “pseudodeficiency” and modifier alleles. In this case report, we describe a child with late-infantile Krabbe disease identified through NBS with markedly reduced GALC activity and a homozygous *GALC* haplotype containing c.956A>G (p.Tyr319Cys; Y319C) and c.1685T>C (p.Ile562Thr; I562T), in addition to other benign variants. Retrospective analysis of the newborn screening specimen demonstrated mild psychosine elevation. Despite preserved neurodevelopment, longitudinal surveillance demonstrated progressive cerebral white matter abnormalities by 3 years and 9 months of age and psychosine elevation in erythrocytes (14 pmol/g Hb; controls < 5), prompting umbilical cord blood transplantation (UCBT). Following transplantation, GALC enzyme activity normalized, psychosine levels decreased, and serial neuroimaging demonstrated radiographic stability without neurologic regression at last follow-up (9 years old). This case expands the phenotypic spectrum associated with homozygosity for the p.Tyr319Cys variant and highlights the role of p.Ile562Thr in amplifying the pathogenic potential when in cis with p.Tyr319Cys. This *GALC* haplotype illustrates how “pseudodeficiency” and modifier alleles may collectively influence biochemical, radiologic, and clinical disease expression. These findings emphasize the importance of integrating genotype, psychosine, enzyme activity, and longitudinal neuroimaging when evaluating infants with NBS results positive for Krabbe disease.

## 1. Introduction

Krabbe disease (OMIM # 245200) is an autosomal recessive leukodystrophy caused by biallelic pathogenic variants in the *GALC* gene, leading to deficiency of galactocerebrosidase (GALC) and subsequent impaired galactolipid degradation and progressive demyelination. In addition to *GALC*, biallelic pathogenic variants in *PSAP* causing Saposin A deficiency (OMIM 611722) may also result in reduced GALC enzyme activity and elevated psychosine concentrations [1]. Clinical manifestations fall on a spectrum. Infantile Krabbe disease (IKD) presents before 12 months of age with symptoms that include extreme irritability, spasticity, global developmental delay, white matter abnormalities on brain imaging and progress to severe neurologic deterioration, with the average age of death around 2 years. Later-onset forms of Krabbe disease (LOKD) present after 12 months of age and are very variable in terms of symptom onset and clinical manifestations [1]. The only available treatment shown to be beneficial is a hematopoietic stem cell transplant (HSCT), which includes umbilical cord blood transplantation (UCBT), and it is recommended that this should be performed for patients with IKD preferably by 30 days of life. To meet such a timeline, newborn screening (NBS) was first initiated for KD in New York state in 2006 by measuring GALC activity followed by sequencing *GALC* when reduced activity was found. Emerging NBS data from NY and subsequently other states suggests that the proportion of individuals with possible LOKD is higher than previously estimated, although to date, very few individuals identified by NBS have developed clinical LOKD [2]. The presence of multiple *GALC* variants with differing classifications, including pseudodeficiency variants, variants of uncertain significance (VUS), and likely pathogenic variants, complicates genotype–phenotype prediction and counseling in asymptomatic newborns identified through screening. Replacement of molecular genetic second-tier testing with measurement of psychosine (galactosylsphingosine) has improved newborn screening, particularly for IKD, which is typically associated with markedly elevated psychosine levels. However, mild to moderate psychosine elevations do not allow for accurate prognostic prediction, particularly when occurring in the setting of complex genotypes that include a VUS or modifier alleles [3].

We describe an infant with late-infantile Krabbe disease (LIKD, symptom onset between 1 and 3 years) identified through NBS with low GALC enzyme activity who was homozygous for three *GALC* variants and was found to have a mild elevation of psychosine in a retrospectively analyzed NBS sample. The proband carried a complex *GALC* genotype consisting of homozygosity for a cis haplotype NM_000153.3:c.[742G>A;956A>G;1685T>C], NP_000144.2:p.[(Asp248Asn;Tyr319Cys;Ile562Thr)]. Identification through newborn screening prompted close longitudinal biochemical and neuroimaging surveillance, ultimately leading to timely UCBT following development of progressively abnormal neuroimaging studies despite preserved neurodevelopment.

## 2. Case Report of Krabbe Disease

### 2.1. Prenatal Course and Newborn Screening Results

The proband was born at 39 weeks and 6 days gestation following an uneventful pregnancy via uncomplicated normal spontaneous vaginal delivery. There were no neonatal complications, and the patient was discharged home from the routine newborn nursery with his mother after a two-day hospitalization.

**Figure 1:**
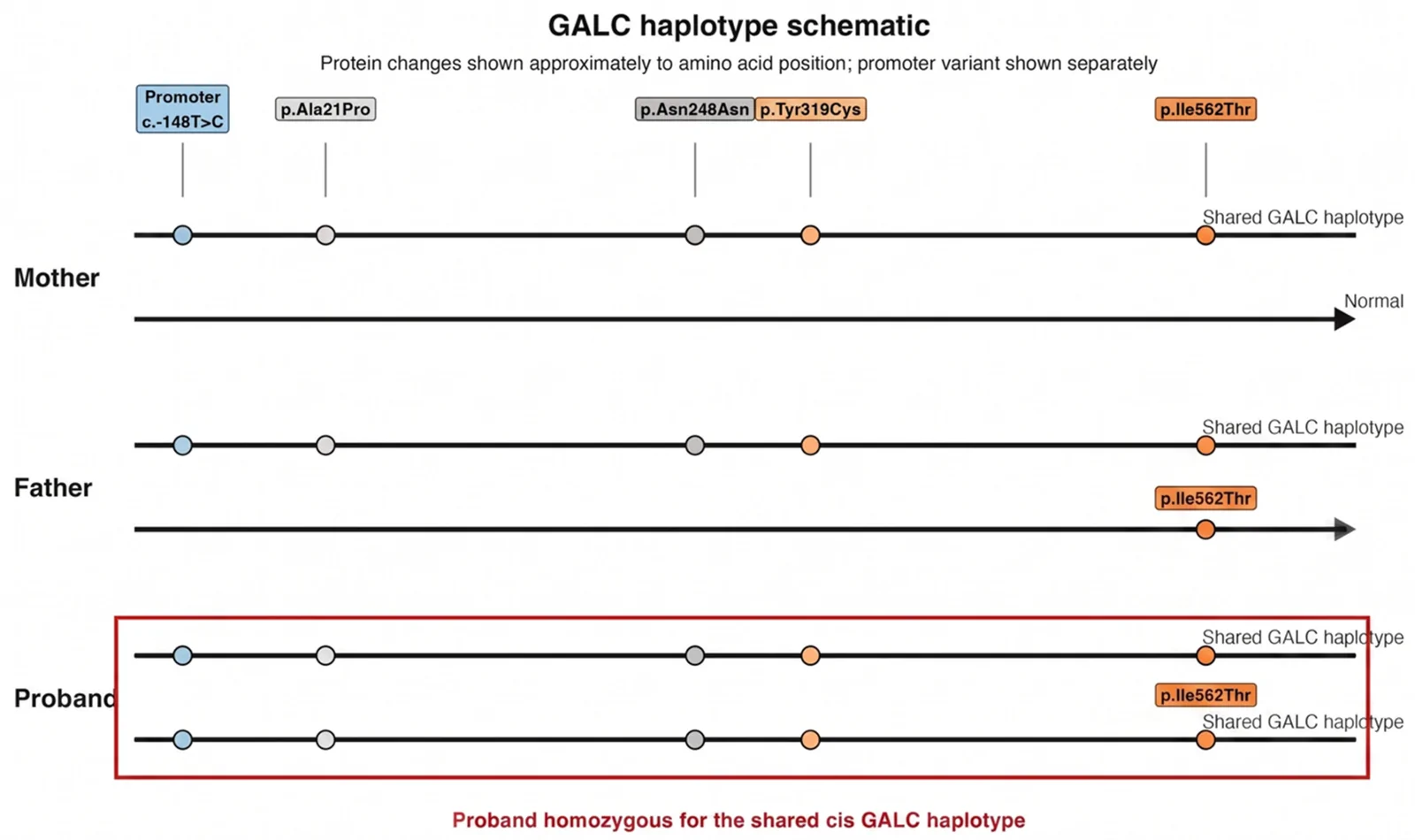
Schematic representation of *GALC* haplotypes in the proband and parents. Both parents carried a shared cis haplotype containing the benign promoter variant c.-148T>C and the coding changes p.Ala21Pro, p.Asn248Asn, p.Tyr319Cys, and p.Ile562Thr. The proband inherited this haplotype from both parents and was therefore homozygous for the shared cis *GALC* haplotype. The schematic is not to genomic or protein scale.

At 12 days of life, the infant was referred to the Division of Medical Genetics for confirmatory testing following a positive NBS for Krabbe disease. The NBS results demonstrated reduced GALC activity in dried blood spots (DBSs) (2.2% of daily mean; controls > 12%) and a complex *GALC* genotype involving homozygosity for five *GALC* variants (Figure 1):

### 2.2. Interpretation of the Genotypes

The proband was homozygous for c.956A>G (p.Y319C; p.Tyr319Cys), which has conflicting classification of VUS or likely pathogenic variant across laboratories. However, more recently, this variant has been associated with disease when in trans with another pathogenic variant, and in vitro functional studies demonstrated reduced GALC activity caused by p.Tyr319Cys [4]. This variant is common in Southeast Asian and Middle Eastern populations and has been associated with LOKD, particularly with visual pathway involvement and vision loss [4]. The earliest reported symptomatic onset associated with this variant occurred at approximately 2.5 years of age in a patient homozygous for p.Tyr319Cys who also carried one copy of the disease modifier variant p.Ile562Thr [4].

The c.1685T>C (p.I562T; p.Ile562Thr) variant is a known polymorphism considered nonpathogenic in homozygous individuals, but it is also considered a “pseudodeficiency” variant because in vitro studies have shown that it reduces GALC enzyme activity [3,4,5]. When p.Ile562Thr is in cis with another milder *GALC* variant, the haplotype effect has been shown to further reduce GALC activity [5,6]. Based on these observations, we hypothesized that p.Ile562Thr, when present in cis with p.Tyr319Cys, may amplify the functional effect of p.Tyr319Cys and contribute to an earlier-onset clinical phenotype.

The patient was also homozygous for c.742G>A (p.Asp248Asn), a common allele considered a pseudodeficiency variant and nonpathogenic in homozygous individuals. There are a few studies showing mild reduction in GALC activity in cells with p.Asp248Asn. However, when p.Asp248Asn is in cis with another milder *GALC* variant, it appears the haplotype effect is an increase rather than a decrease in GALC activity [7]. Finally, the patient was homozygous for c.-148T>C, a benign promoter variant.

For our proband, both parents were heterozygous for all variants (Figure 1).

### 2.3. Initial Assessment and Family History

During the initial assessment, physical examination was normal without any neurological abnormalities, and confirmatory testing showed GALC enzyme activity in leukocytes of 0.03 nmol/h/mg protein, within the high-risk range for Krabbe disease (0.0–0.15) (Lysosomal Diseases Testing Laboratory, Thomas Jefferson University, Philadelphia, PA, USA) [5,6].

Family history was significant for consanguinity, as the parents are first cousins of Southeast Asian descent. The paternal uncle, paternal aunt, and father all reportedly had a neurodegenerative condition characterized by ataxia, tremor, and cognitive decline with onset in the early third decade of life. Subsequent studies showed that the father, paternal uncle and paternal aunt were homozygous for a pathogenic variant in *SPG11*, and their symptoms were consistent with Hereditary Spastic Paraplegia 11.

The family was counseled regarding the broad clinical spectrum of Krabbe disease in the proband and the need for biochemical and neuroimaging surveillance.

### 2.4. Early Neurologic Evaluation and Neuroimaging Findings

At five weeks of age, the infant was admitted for 3 days for comprehensive baseline evaluation, including video electroencephalography (vEEG), brain magnetic resonance imaging (MRI) with magnetic resonance spectroscopy (MRS), and lumbar puncture. The vEEG demonstrated normal background activity without epileptiform discharges. Brain MRI and MRS were within normal limits. Cerebrospinal fluid analysis revealed normal glucose, sterile cultures, normal cell counts and immunoglobulin levels, and only mildly elevated protein at 67 mg/dL (NI 15–45 mg/dL); oligoclonal bands were absent. Overall, the findings were not suggestive of demyelination or active neuroinflammation.

**Figure 2:**
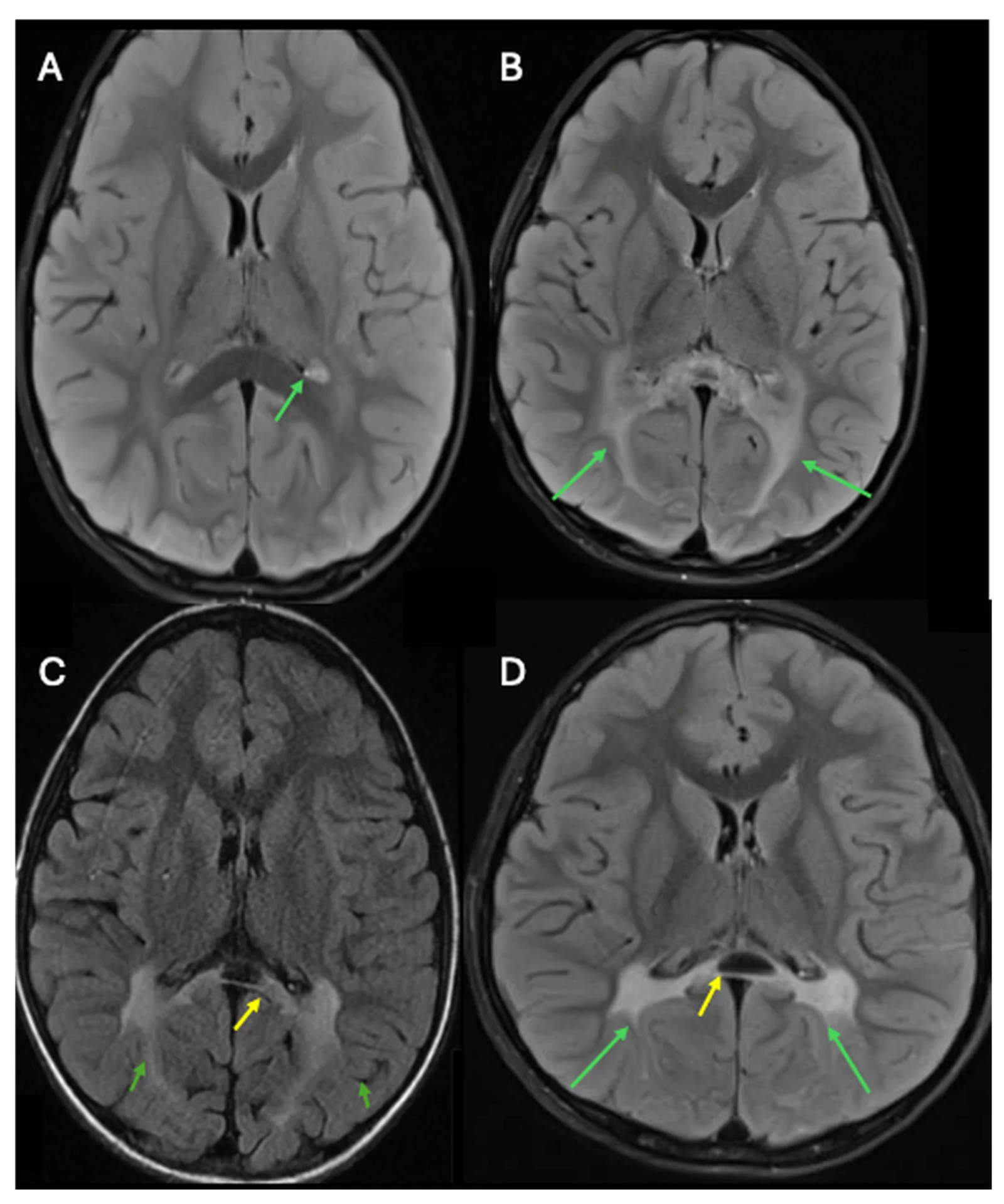
Longitudinal brain MRI demonstrating the evolution of white matter abnormalities in a patient with Krabbe disease. Axial FLAIR imaging obtained at two-and-a-half years of age demonstrates mild, symmetric bilateral periatrial white matter T2/FLAIR hyperintensity (green arrows), which was of uncertain significance and may represent either a normal variant or early manifestation of Krabbe disease (**A**). Follow-up axial FLAIR imaging obtained around three-and-a-half years of age demonstrates progression of T2/FLAIR hyperintensity involving the posterior periatrial white matter and forceps major (green arrows) (**B**). Axial T2/FLAIR imaging at 6 years of age, approximately two years following the second transplant, demonstrates stable moderate T2 hyperintense signal within the posterior centrum semiovale and corona radiata (green arrows), with interval development of cystic gliosis involving the splenium of the corpus callosum (yellow arrow) (**C**). Most recent axial T2/FLAIR imaging obtained at 9 years of age demonstrates stable periatrial T2/FLAIR hyperintensity involving the splenium of the corpus callosum (green arrows) and persistent cystic gliosis within the splenium (yellow arrow) (**D**).

Serial MRI studies were subsequently obtained to monitor early leukodystrophic changes and were normal at 15 months and at 27 months. Due to the COVID-19 pandemic, the next MRI was delayed until the patient was 3 years and 9 months of age. Findings included new abnormalities, including increased Fluid-Attenuated Inversion Recovery (FLAIR) signal in the parietal and periventricular white matter, new splenial involvement, and signal abnormalities involving the cerebral peduncles, lateral midbrain, and pons, as well as patchy T2 hyperintensity in the cerebellar white matter surrounding the fourth ventricle (Figure 2). Concurrent spine MRI revealed patchy T2 hyperintensity and expansion of the cervical spinal cord extending from the cervicomedullary junction to C7, with focal involvement of the posterior columns, consistent with leukodystrophic spinal cord involvement.

The patient’s globoid cell leukodystrophy (GLD) MRI score was 9, with regional involvement distributed as follows: periventricular and subcortical white matter (3 points), corpus callosum (1 point), visual pathways (2 points), pyramidal system (2 points), and cerebellum (1 point) [8]. The GLD/Loes MRI scoring system ranges from 0 (normal) to 32 (severe disease burden), with higher scores reflecting increasing leukodystrophic involvement; thus, a score of 9 is consistent with established radiographic leukodystrophy [9,10]. Neurologic examination remained normal apart from mild appendicular hypotonia, and the functional motor score via the Gross Motor Function Classification in Metachromatic Leukodystrophy was normal, 0 [11].

Psychosine was measured in erythrocytes and found to be elevated (14 pmol/g Hb; controls < 5; Biochemical Genetics Laboratory, Mayo Clinic, Rochester, MN). Given the abnormal neuroimaging studies and elevated psychosine, the patient was referred for umbilical cord blood transplantation (UCBT) as definitive therapy.

### 2.5. Transplant Management

UCBT was performed shortly before the proband’s fourth birthday using an unrelated 6/8 HLA-matched umbilical cord blood donor. Engraftment was achieved by day +15. Graft-versus-host disease (GVHD) prophylaxis consisted of mycophenolate mofetil (MMF) and cyclosporine A. At six months, peripheral blood chimerism demonstrated 100% donor contribution across all lineages except for T cells (7%).

**Figure 3:**
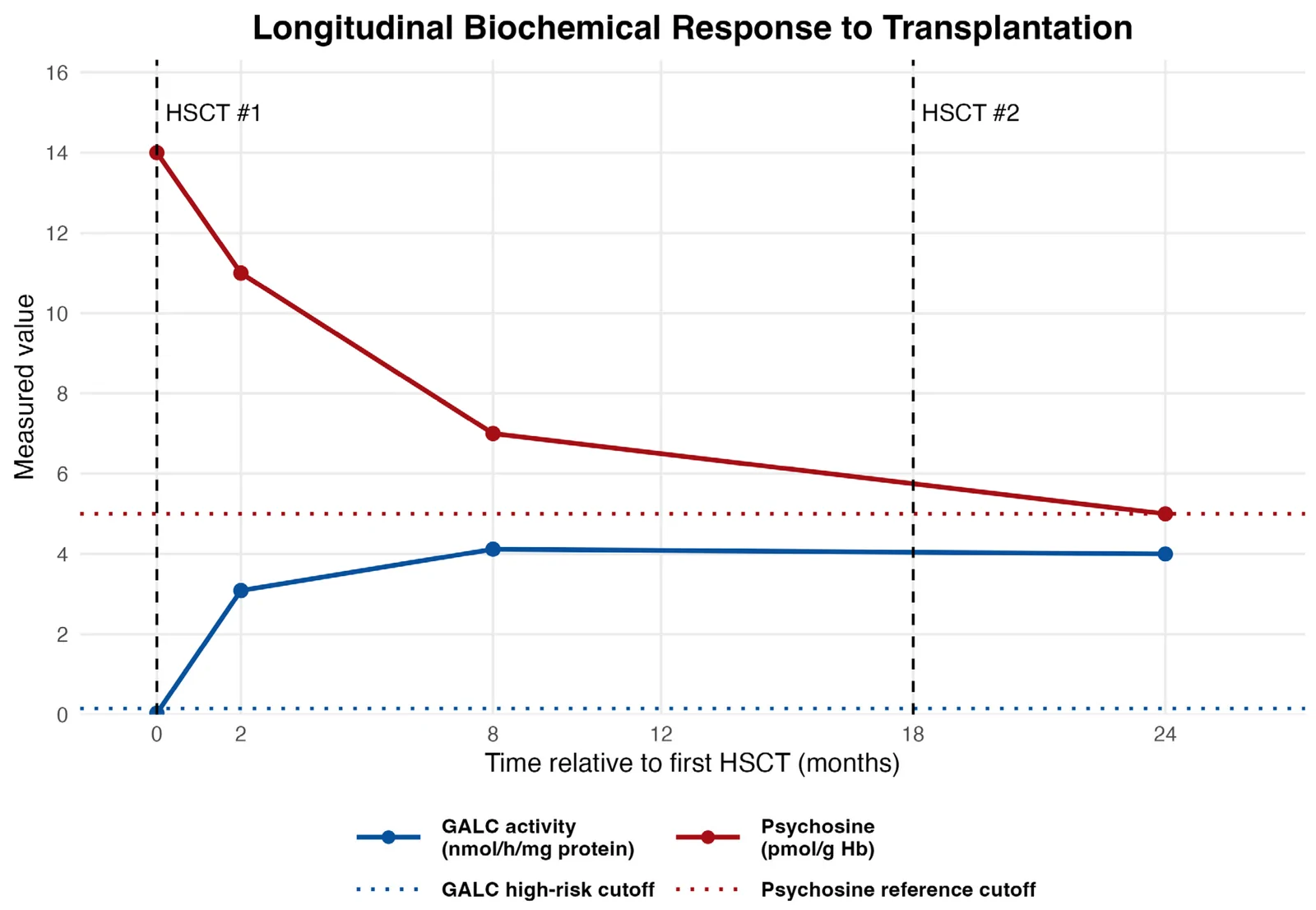
Longitudinal biochemical response to hematopoietic stem cell transplantation in a patient with Krabbe disease. GALC enzyme activity increased following transplantation, while psychosine levels declined over time. The blue dotted line indicates the GALC activity high-risk cutoff (0.15 nmol/h/mg protein), and the red dotted line indicates the psychosine reference cutoff (5 pmol/g Hb). Vertical dashed lines indicate timing of hematopoietic stem cell transplants. Together, these findings demonstrate biochemical correction and stabilization following treatment.

Biochemical monitoring following UCBT showed improved disease biomarkers (Figure 3):

- GALC enzyme activity: 0.03 nmol/h/mg (pre-UCBT) → 3.09 nmol/h/mg (2 months post-UCBT) → 4.12 nmol/h/mg (8 months post-UCBT).
- Psychosine in erythrocytes: 14 pmol/g Hb (1-month pre-UCBT) → 11 pmol/g Hb (2 months post-UCBT) → 7 pmol/g Hb (8 months post-UCBT).

**Figure 4:**
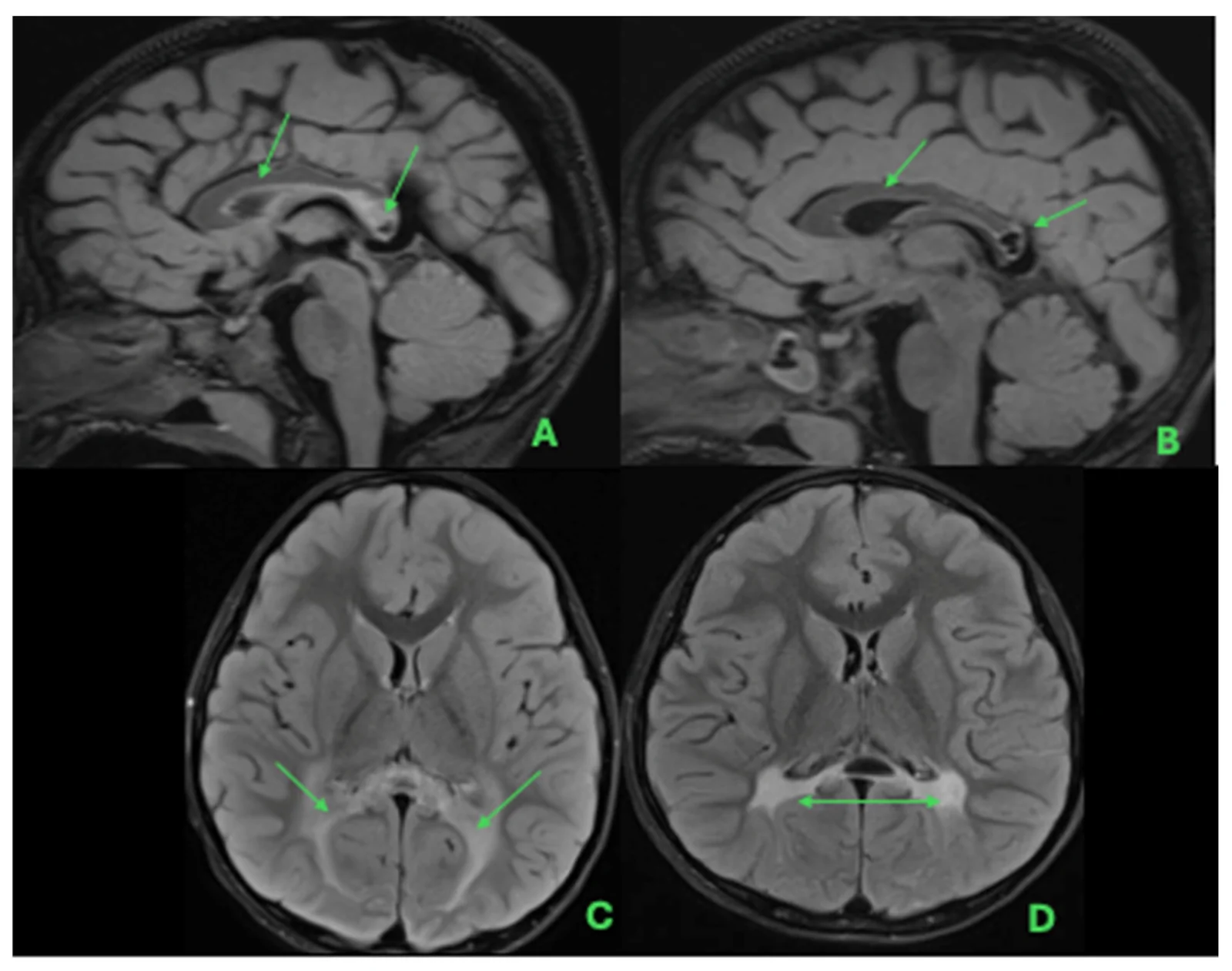
Serial brain MRI demonstrating pre- and post-transplant evolution of white matter abnormalities in a patient with Krabbe disease. Pre-transplant axial T2 FLAIR imaging demonstrates involvement of the splenium of the corpus callosum (**A**) and symmetric extension of abnormal signal within the bilateral periatrial and parieto-occipital periventricular white matter (**C**). Follow-up imaging after the second transplant demonstrates stable involvement of the corpus callosum on sagittal FLAIR sequences (**B**), with persistent but stable periventricular white matter abnormalities on subsequent imaging approximately one year later (**D**).

Follow-up imaging showed stabilization of white matter abnormalities on serial MRI (Figure 4). Seven weeks post-UCBT, brain MRI showed slightly accentuated posterior periventricular white matter signal with mild cerebral sulcal prominence suggestive of early atrophy; spine MRI was unchanged. One year post-UCBT, brain MRI demonstrated decreased posterior periventricular white matter abnormalities and overall stable findings, indicating interval improvement.

Approximately 17 months after the transplant, donor chimerism declined to 0%, indicating autologous reconstitution. The post-transplant course was further complicated by autoimmune cytopenias (and may have played a role in late graft rejection), which initially responded well to steroids and Rituximab, and, early after transplant, also stage 1 gastrointestinal (overall grade 2) GVHD, responding well to steroid therapy.

Given the eventual loss of donor chimerism, the patient underwent a second UCBT using an unrelated 5/8 HLA-matched umbilical cord blood donor 18 months after the initial procedure. Conditioning therapy included fludarabine, treosulfan, and low-dose (200cGy) total-body irradiation. GVHD prophylaxis comprised tacrolimus and MMF. The latter was discontinued due to transient hyperkalemia. Engraftment occurred by day +13, and follow-up MRIs have demonstrated stable to mildly improved white matter signal abnormalities with no new lesions. Without major complications after the second transplant, the patient remains full donor chimeric in myeloid and T cell compartments at the latest follow-up (2+ years after the second transplant).

### 2.6. Development

At the most recent follow-up at 9 years old, the patient demonstrated normal cognitive, language, and motor development for age, with no history of developmental regression. Neurologic examination remained normal apart from baseline mild appendicular hypotonia. There have been no seizures, and follow-up EEGs have remained normal. Psychosine levels in erythrocytes have significantly decreased from the pre-transplant level of 14 pmol/g Hb to mildly elevated levels around 7 pmol/g Hb, while GALC enzyme levels in leukocytes remain within the normal range, and serial brain MRIs reveal no evidence of disease progression.

### 2.7. Discussion

This case highlights a complex *GALC* genotype identified through newborn screening, in which the proband was homozygous for both p.Tyr319Cys and p.Ile562Thr variants. Each allele has been independently described in homozygosity, but homozygosity for both variants is a novel observation. While p.Tyr319Cys has been associated with LOKD and p.Ile562Thr is typically considered benign or low-penetrance when present in isolation, this unique combination provides new insight into the spectrum of Krabbe disease. It illustrates how modifier effects can complicate genotype–phenotype interpretations in asymptomatic infants and provides valuable insight into how this genotype may influence the biochemical phenotype, radiologic findings, and overall disease expression.

Homozygosity for the p.Tyr319Cys variant has been associated with LOKD, most often presenting with isolated ocular involvement, specifically severe or complete vision loss [4]. The earliest reported symptomatic onset for individuals harboring this variant has been around 2.5 years of age in a patient who was compound heterozygous for p.Tyr319Cys and a more severe *GALC* variant [3]. A 2020 case series reports three individuals of Asian descent with pediatric-onset Krabbe Disease and vision loss [4]. However, a prior publication based on NBS data from New York identified individuals homozygous for p.Tyr319Cys, with low enzyme activity but without Krabbe disease manifestations at the time of reporting [12].

Interpretation of the pathogenicity of the *GALC* p.Tyr319Cys variant varies greatly between laboratories per the ClinVar database [13]. Of the 21 submissions in ClinVar as of January 2026, 10 submitters considered the variant to be pathogenic/likely pathogenic, eight to be of uncertain significance, and three as likely benign/benign. Of the submissions that classified the variant as likely benign or benign, one institute describes that the classification primarily stems from the Benign Strong (BS1) criterion for classifying variants per ACMG guidelines [1]. Per these submissions, suspicion for the pathogenicity of this variant is reduced, as this variant’s frequency in population databases is higher than expected for a rare disease. Per the population database gnomAD, this variant has been identified in >1% of individuals of South Asian descent [14]. While this allele frequency is high, this criterion does not take into consideration that patients with LOKD may not present to care in the same manner as patients with IKD or undergo genetic testing for their manifestations. As such, while the allele frequency is high, we also cannot rule out that there may be a higher incidence of affected individuals with LOKD as well. Of note, the variability in disease associated with p.Tyr319Cys may be due to some patients also carrying the p.Ile562Thr variant in cis, which may not have been reported routinely by clinical laboratories because, by itself, it is a non-disease-causing variant.

The p.Ile562Thr variant is well-described and is considered nonpathogenic in homozygous individuals [1]. However, functional and population data indicate that p.Ile562Thr can act as a modifier allele, affecting GALC enzyme folding or trafficking when present in cis with a pathogenic variant [1,15]. It has been proposed that the p.Ile562Thr allele may amplify the pathogenic potential of p.Tyr319Cys when co-occurring in cis, producing a more severe biochemical phenotype despite clinical stability [7,16]. To date, however, no individuals homozygous for both p.Tyr319Cys and p.Ile562Thr in cis have been reported, and the extent of this modifier effect remains poorly understood.

In this proband, combined homozygosity for p.Tyr319Cys, together with the pseudodeficiency variant p.Ile562Thr and p.Asp248Asn, resulted in markedly reduced GALC enzyme activity and mildly elevated psychosine levels, consistent with a biochemical profile concerning for Krabbe disease. Longitudinal evaluation demonstrated progressive leukodystrophic changes on MRI that prompted early UCBT, with subsequent normalization of GALC activity, reduction in psychosine levels and radiographic stability.

This case report makes a strong case for the reclassification of the *GALC* p.Tyr319Cys variant from previous classifications as a variant of uncertain significance to likely pathogenic. Given our growing understanding of variants and their contributions to Krabbe disease, the *GALC* p.Tyr319Cys variant may best be described as a “risk allele”, especially when in cis with p.Ile562Thr. However, reports of this variant in individuals affected with Krabbe disease indicate that the variant is neither benign nor of uncertain significance.

Additionally, this case demonstrates how the severity of the Krabbe disease phenotype may be modified by the presence of the p.Ile562Thr variant occurring in cis with p.Tyr319Cys, given the necessity for transplantation in this pediatric patient with a previously presumed later-onset genotype. In this proband, combined homozygosity for p.Tyr319Cys and p.Ile562Thr was associated with markedly reduced GALC activity, persistent psychosine elevation, and progressive radiologic abnormalities despite initially normal neurologic examinations and preserved neurodevelopment. An important lesson from this case and other publications is that investigation for the presence in cis of the p.Ile562Thr allele is critical when the p.Tyr319Cys is identified because the haplotype is a risk allele associated with disease.

As knowledge of the molecular basis of Krabbe disease grows, similar modifier effects may prove relevant for other variants or pseudodeficiency alleles within *GALC*. The significance of this finding lies in its potential to clarify how common or polymorphic alleles can interact to modulate disease risk and influence biochemical and radiologic disease expression.

This case underscores the importance of interpreting NBS results in an integrated framework that incorporates molecular configuration (cis/trans phase), enzyme assays, biomarkers such as psychosine, and longitudinal neuroimaging rather than relying on genotype classification alone. Of note, psychosine was not part of the NBS strategy when this patient was screened; however, retrospective analysis of the original NBS sample revealed a mild elevation of 3.8 nmol/mL (controls < 2.0). Identification of infants homozygous for this combined *GALC* haplotype, together with elevated psychosine levels, should prompt careful longitudinal surveillance. Therefore, individuals undergoing clinical assessment for Krabbe disease should undergo full gene sequencing of *GALC* to evaluate both pathogenic variants and pseudodeficiency or polymorphic variants such as p.I562T that may act as modifier variants. This is also important from a family planning perspective, as commercially available expanded carrier screening may not report pseudodeficient or polymorphic variants of variable clinical significance, as they are considered benign. Greater understanding of the role of these variants will help expand the recognized phenotypic spectrum of Krabbe disease and may improve future prediction of disease severity. Continued follow-up of similar patients will be critical for defining penetrance, modifier interactions, and counseling implications for affected families.

## Data Availability

No new data were created or analyzed in this study. Data sharing is not applicable to this article.

## Abbreviations

NBS	newborn screening
IKDe	Infantile Krabbe disease
NSVD	normal spontaneous vaginal delivery
GALC	galactocerebrosidase
VUS	variant of uncertain significance
vEEG	video electroencephalography
MRI	magnetic resonance imaging
MRS	magnetic resonance spectroscopy
GLD	globoid cell leukodystrophy
UCBT	umbilical cord blood transplantation
GVHD	graft-versus-host disease
MMF	mycophenolate mofetil
HSCT	hematopoietic stem cell transplant
